# Is there still a need for dental textbooks when doubts can be clarified by artificial intelligence and open access science?

**DOI:** 10.3389/fdmed.2023.1236861

**Published:** 2023-08-14

**Authors:** João Paulo Mendes Tribst, Amanda Maria de Oliveira Dal Piva, Cornelis J. Kleverlaan, Albert J. Feilzer

**Affiliations:** ^1^ Department of Reconstructive Oral Care, Academic Centre for Dentistry Amsterdam (ACTA), Universiteit van Amsterdam and Vrije Universiteit, Amsterdam, Netherlands; ^2^ Department of Dental Materials Science and Department of Reconstructive Oral Care, Academic Centre for Dentistry Amsterdam (ACTA), Universiteit van Amsterdam and Vrije Universiteit, Amsterdam, Netherlands

**Keywords:** artificial intelligence, ChatGPT, dentistry, clinical practice, ethics, language models

## Landmark

A groundbreaking novelty in artificial intelligence (AI) marked November 2022: Chat Generative Pre-trained Transformer (ChatGPT) was released. In the dental field, AI was classified as a user-friendly, transparent, reproducible, and unbiased adjunct for dentists (1). However, ChatGPT goes beyond that and, according to users' prompts, can write emails, computer codes, and even multiple choice exam questions. Do we face a disruptive change by its birth?

Researchers started merging worry with their excitement, considering a potential impact on human knowledge and scientific ability, and citing the challenge of discriminating between human-written and AI texts (2–5). Not everything about the forthcoming changes is negative: academics from health sciences should be aware that ChatGPT is a cutting-edge AI language model, however it is not flawless (2). With feedback it learns to improve text generation and can be trained to use massive (publicly accessible) online data, providing information resemblant to academic publications, overcoming the limitation that without open access many academic publications are not readily accessible to those seeking information.

The concept of “garbage in, garbage out” (GIGO) means that the quality of output from a system is dependent on the quality of input data. If the user inputs flawed or incorrect information, the results will also be flawed or inaccurate. This principle applies to the AI language models as well. While ChatGPT is designed to generate coherent responses based on its training data, it is not immune to the limitations of GIGO. Ultimately, the responsibility for the quality of output lies with the users (3). ChatGPT answer should be a new topic in the education of undergraduate students: learning to judge the reliability of artificial intelligence, and comparing the quality with a published scientific manuscript.

One of the most attractive advantages of ChatGPT is that it offers a conversational, interactive platform for users and provides direct responses to queries instead of directing the user to numerous websites. According to the bot, it can assist students and researchers in finding relevant sources for their research, as well as providing summaries of academic papers and scientific articles. It can also help with literature reviews and citation management, providing explanations of complex concepts, definitions of key terms, and examples of how to apply theories and models in different contexts. For non-native English speakers, ChatGPT can help with grammar and language issues in academic writing, and provide language support for international students who may need help adjusting to a new academic environment. Additionally, ChatGPT can offer personalized learning experiences, adapting to the unique needs and preferences of individual learners. It can provide feedback on assignments and help students identify areas where they need additional support. Artificial intelligence can assist with exam preparation by providing practice questions, quizzes, and other study materials. It can also help with test-taking strategies and time management skills. The possibilities are limitless, but more important than what this tool can do is how we are going to use and adapt to it ourselves.

## Prospects in dental education

In dentistry, will we be able to use AI-text generators in our favor? We previously faced well-informed patients who were often confused by the mass of internet data and open access science. Now, this tool can informally answer their questions on a trustable level in a short report.

A recent investigation assessed clinical and educational aspects of dentistry with practical applications of using ChatGPT as an artificial intelligence tool. According to the authors, the evolution of courses at dental schools is inevitable, as advanced deep-learning approaches take over the clinical areas of dentistry, redesigning diagnostics, treatment planning, organization, and teledentistry (5). With recent advances in these artificial language models, interaction with patients will also be modified, and the basis of dental education—including dissertations, theses, and writing scientific papers—will need to be revised (5). Because internet access is readily available, patients, students, dental technicians, and dentists can immediately access online information; the reported suggestions are reasonable and exciting.

ChatGPT or AI bots cause heated discussion between progressivism and conservatism in the academic environment, but soon it will be as common as text editors in the university environment (5). In this scenario of fluid future prospects in dental education, a preprint article investigated the impact of using ChatGPT on common assessments in dental education. Their results demonstrate plenty of scope for ChatGPT to attempt dental assessments and achieve acceptable grades (6). The potential for providing reliable answers to common questions may also replace traditional web search engines, saving time and allowing an improved filter from the vast ocean of information, advertisements and distractions that can be found online.

In addition to the powerful and wide range of applicability offered by artificial intelligence systems, other parameters dependent on institutional factors, leadership, resource, systems, and culture will modify the vision of accessibility, inclusive teaching, and student support (7). To be considered truly inclusive, the use of artificial intelligence should be also associated with open access, requiring a whole institution approach in the educational field. This can be particularly difficult because such modifications would probably impact the working practices, management of resources, and competences. This laborious process would be reflected in students not only being able to solve their own questions, but to search and prove their point with up-to-date free access data. A tool offering auto-generated answers can only be trusted if auditable.

The practice of making scientific research and data freely available to the public, without any cost or subscription barriers, is becoming increasingly important in today's world, for the following reasons:

• Increased accessibility: Open access science allows anyone, regardless of their financial or institutional status, to access scientific research and data. This promotes equity and allows for broader participation in scientific discussions and discoveries.
• Accelerated progress: By making research more accessible, open access can accelerate scientific progress. When more people have access to scientific findings, they can build on those findings and generate new insights more quickly.
• Greater collaboration: Open access science can facilitate collaboration between researchers, leading to more diverse perspectives and greater innovation.
• Enhanced transparency: By making research findings and data publicly available, open access science can promote greater transparency and accountability in scientific research.
• Increased impact: Open access can increase the impact of scientific research by making it more widely known and cited. This can lead to greater recognition for researchers and their work, and can help to attract more funding for future research.

Despite the accepted need for more openness and innovation, in practice, scientific education remains essentially closed and fragmented. This is also a reflection on research evaluation practices based on the number of citations and journal impact factors, scientific strategies focused on profit, unequal information distribution, and lack of adequate peer-review process (8, 9). Therefore, it is not only important to make the science available for everyone, but also to determine in which databases the scope fits best; as well as how to separate the wheat from the chaff when reading a freely-available manuscript. In summary, open science is quickly growing and will broaden our horizons in terms of innovation, with significant implications for society, policy, and numerous academic research areas (8). When facing global challenges, higher education programs need to control different stakeholder groups and find new ways to manage students thinking like scientists, the importance of critical thinking and how the use of artificial intelligence can be positively associated with that.

**Figure 1:**
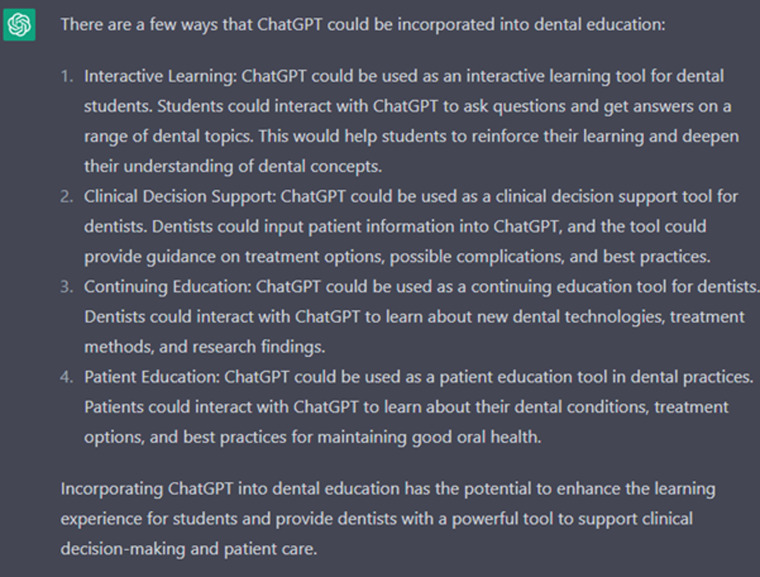
Suggestion provided by ChatGPT on how to incorporate it in the field of dental education.

It is expected that AI language models, e.g., ChatGPT, do not have the ability to directly influence the availability of scientific research and data. However, ChatGPT can still support the principles of open access by promoting awareness of the benefits of open access, and by providing information and resources to researchers and the public. Despite being challenging, the bot can help when questioned about “How can ChatGPT be incorporated into dental education?”. The answer is showed in Figure 1.

The provided answer is not far from the reality of its current application when considering a single user, an institution, and even a global application, yet it demands effort to incorporate this tool in the dental field.

While traditional textbooks can still be a valuable resource in many educational settings, educators and students are increasingly turning to alternative sources of information and learning resources to supplement or replace textbooks. In brief, textbooks falling out of use can be a result of outdated content, high cost, digital learning, customized curriculum, or alternative teaching methods. Until now, there are many alternative sources of information that can be used instead of textbooks, including online resources, open educational, library resources, peer-reviewed articles, podcasts, and videos. Students and patients are aware of that.

**Figure 2:**
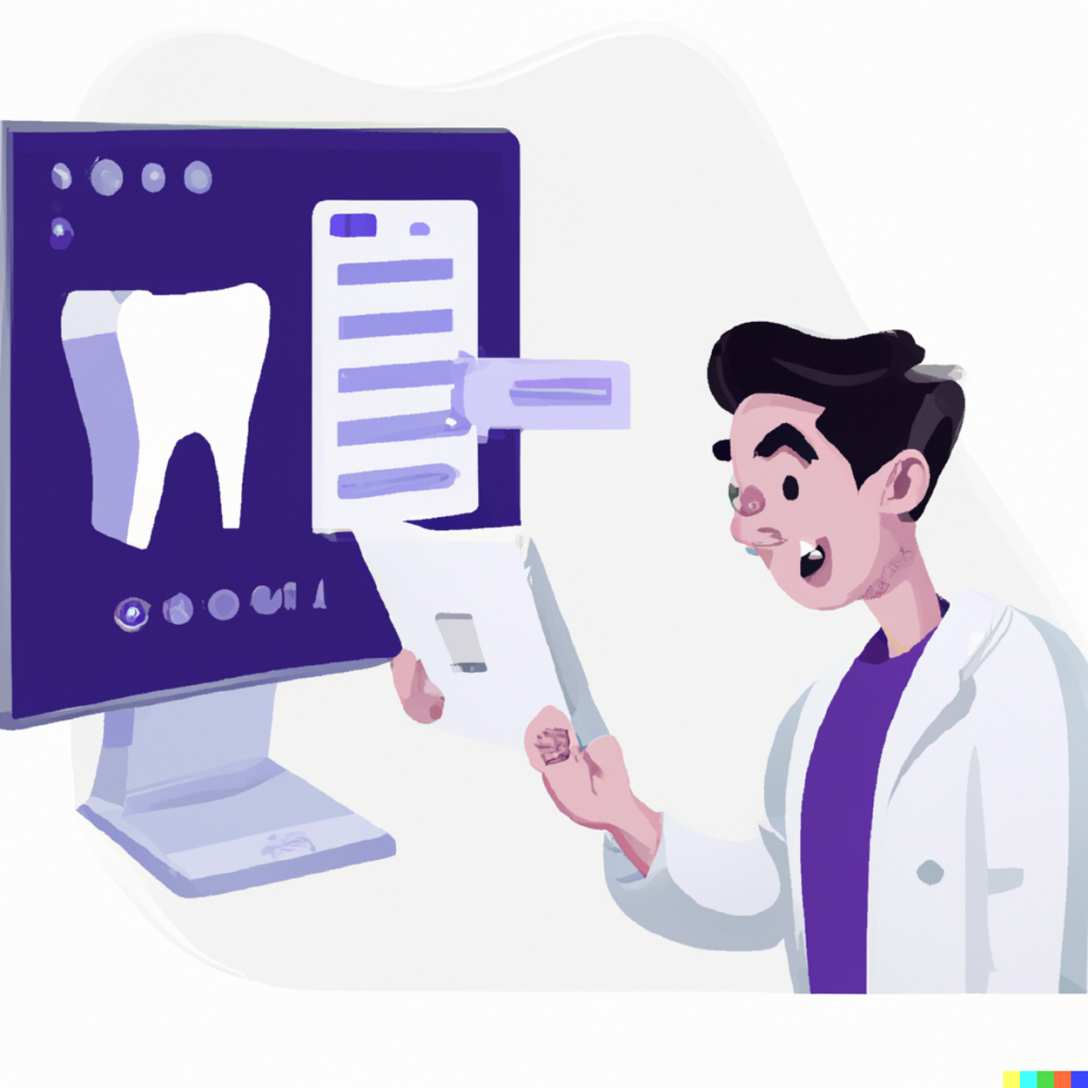
Digital image generated by the OpenAI DALL-E 2, based on the text “Dentist using AI-text generator to interpretate an open access article”.

This paradigm shift highlights the increasing demand for applying robust AI guidelines in teaching and preparing yourself to talk with patients and students (Figure 2). As academics, instead of being afraid, we need to embrace AI tools in our favor and prepare our students for a new type of learning: possibly with fewer textbooks. We should not disregard AI to get information since this letter was partially written by it.
